# Resveratrol ameliorates autophagic flux to promote functional recovery in rats after spinal cord injury

**DOI:** 10.18632/oncotarget.23877

**Published:** 2018-01-03

**Authors:** Peng Wang, Lizhu Jiang, Nian Zhou, Hao Zhou, Huzhe Liu, Wenrui Zhao, Hanxiang Zhang, Xiang Zhang, Zhenming Hu

**Affiliations:** ^1^ Department of Orthopedic Surgery, The First Affiliated Hospital of Chongqing Medical University, Chongqing, 400016, China; ^2^ Department of Otorhinolaryngology Surgery, The First Affiliated Hospital of Chongqing Medical University, Chongqing, 400016, China

**Keywords:** resveratrol, spinal cord injury, autophagy flux, apoptosis, LKB1/AMPK/mTOR/p70s6k pathway

## Abstract

Resveratrol is known to improve functional recovery after spinal cord injury, but the exact mechanism involved is yet unclear. The aim of this study was to clarify whether resveratrol can exert neuroprotective effects via activating neuronal autophagic flux, in view of the underlying role of the autophagic flux mediated by resveratrol on neuronal apoptosis after spinal cord injury, and identify the role of the liver kinase B1(LKB1)/adenosine monophosphate-activated protein kinase (AMPK)/mammalian target of rapamycin (mTOR)/ p70 ribosomal protein S6 kinase (p70s6k) signal pathway in the autophagic flux mediated by resveratrol. The results obtained strongly indicate that resveratrol improved functional recovery in Sprague–Dawley rats after acute spinal cord injury, preserved their motor neurons, alleviated the neuronal apoptosis, and ameliorated neuronal autophagic flux. After blocking the autophagic flux, the neuroprotective effects of resveratrol were eliminated. Furthermore, it was proved that resveratrol can activate the LKB1/AMPK/mTOR/p70s6k pathway *in vivo* and *in vitro*, and the LKB1/AMPK/mTOR/p70s6k pathway plays a vital role in activating the autophagic flux mediated by resveratrol in PC12 cells. Thus, resveratrol enables to ameliorate neuronal autophagic flux via the LKB1/AMPK/mTOR/p70s6k pathway to alleviate apoptosis, and finally ameliorating functional recovery after acute SCI in SD rats.

## INTRODUCTION

Spinal cord injury (SCI) is a highly serious disease of the central nervous system, which leads to the destruction of the motor and/or sensory function resulting in temporary or permanent disability. There are about 273000 SCI patients in the United States, with 12000 new SCI cases annually [1]. However, there is no effective therapy to treat SCI in clinic up to now. Thus, the disease has brought great challenges to both clinical and scientific researchers. SCI includes two phases of pathology: the primary and secondary injuries [2]. The primary injury, which is irreversible, means that the spinal cord is injured by a direct or indirect violence. However, the secondary injury, following the primary injury, causes a sustained injury at the molecular and cellular level surrounding the epicenter of the lesion, including edema, hemorrhage, apoptosis, inflammation and oxidative stress, significantly affecting the prognosis of SCI [3–5]. It is considered as an effective method to treat SCI by reducing the secondary injury. Although the exact mechanism is still unclear, apoptosis plays a major role in the secondary injury. Previous studies have shown that inhibiting apoptosis can lead to improvement of the prognosis of SCI [6]. Thus, attenuating or blocking apoptosis may improve the rehabilitation from SCI.

Recently, more and more studies have paid close attention to the role of autophagy in the central nervous system injury [7, 8]. Autophagy, as an important and conserved lysosomal degradation pathway, is considered to be involved in many physiological and pathological situations and to play a major role in maintaining cellular homeostasis by disposing of the damaged proteins and organelles with energy reuse or production [9, 10]. It is shown that the stimulated autophagy can inhibit apoptosis to recover the neurological function in diabetic SCI rats [11]. On the other hand, some researchers reported that the inhibited autophagy could exert neuroprotective effects [12, 13]. Consequently, the role of autophagy in SCI appears to be disputed. This dispute is associated with autophagic flux, which is a dynamic process, including the formation, delivery, and degradation of autophagosomes. However, an accumulation of autophagosomes may occur due to a reduction in autophagosome degradation or inability of degradation to follow the increased autophagosome formation. The beneficial or detrimental function of autophagy may depend on induction or inhibition of autophagic flux after injury of the central nervous system, with unobstructed autophagic flux often leading to cytoprotection and the obstructed autophagic flux contribution to cell death [14]. Thus, modulating autophagic flux may be beneficial for the treatment of SCI.

Resveratrol (3,5,4′-trihydroxy-trans-stilbene) is a type of natural phenol, and a phytoalexin produced by several plants in response to injury or when the plant is under attack by pathogens such as bacteria or fungi. Sources of resveratrol in food include the skin of grapes, blueberries, raspberries, mulberries, lingonberries, and senna [15]. Resveratrol also offers a broad range of health advantages, including neuroprotection [16–21]. Recently, there is an increasing evidence of resveratrol capacity to improve the motor function of hind limbs after SCI [22, 23]. However, the exact mechanism involved is still unclear. The available evidence demonstrated that resveratrol could promote autophagic flux to protect dopaminergic neurons [16]. Our previous study has also shown that resveratrol can promote autophagy to protect the degenerative human disc nucleus pulposus cells [24]. This evidence revealed that autophagic flux might be involved in cytoprotection of resveratrol. However, whether resveratrol can exert neuroprotective effects via activating neuronal autophagy flux remains unclear, and the underlying role of the resveratrol-mediated neuronal autophagic flux on neuronal apoptosis after SCI in the laboratory SD rats needs to be clarified.

Numerous studies have shown that AMPK/mTOR/p70s6k pathway plays a major role in neuroprotection via enhancing autophagy [25]. AMPK, as the main controller of cellular energy homeostasis, can regulate the energy balance through different signal pathways [26, 27]. Also, mTOR/p70s6k pathway can negatively affect the autophagy [28]. since the activation of AMPK for inhibiting of the mTOR/p70s6k pathway was reported to enhance the autophagic flux [29]. LKB1 could also activate AMPK and be involved in the subsequent autophagy induction [30]. However, the role of LKB1/AMPK/mTOR/p70s6k signaling pathway in the resveratrol-mediated autophagic flux is yet unclear and needs to be investigated.

To resolve this issue, we designed the study to determine whether resveratrol improves the motor function of hind limbs via enhancing neuronal autophagic flux, and the underlying role of the resveratrol-mediated neuronal autophagic flux on neuronal apoptosis after SCI in our experiment on SD rats. Furthermore, we also investigated the involvement of the LKB1/AMPK/mTOR/p70s6k signaling pathway in the autophagic flux mediated by resveratrol.

## RESULTS

### Resveratrol improves the motor function of hind limbs and preserves the motor neurons after acute SCI in SD rats

**Figure 1:**
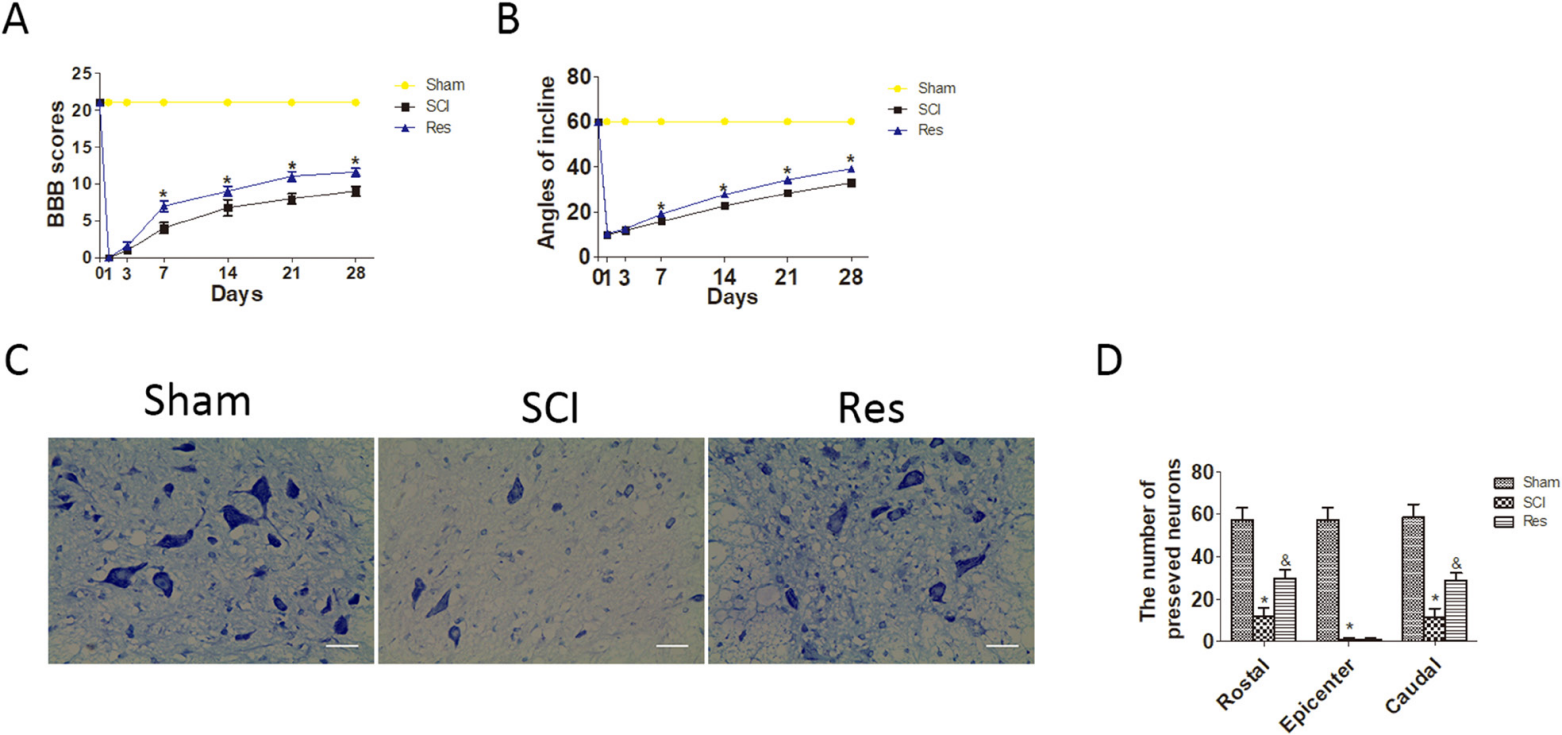
Resveratrol (Res) improves the motor function of hind limbs and the number of the preserved motor neurons after acute SCI in SD rats (**A**) The BBB scores of rats in sham, SCI, and Res groups in 1, 3, 7, 14, 21 and 28 days after the injury. ^*^P < 0.001 versus the SCI group. *n* = 5 per group. (**B**) The inclined plane test scores in sham, SCI, and Res groups in 1, 3, 7, 14, 21, and 28 days after the injury. ^*^P < 0.001 versus the SCI group, *n* = 5 per group. Values are expressed as the mean ± SD, *n* = 5 per group. (**C**) Nissl staining of the sham group, SCI group and Res group at seven days. Scale bars are 20 μm. (**D**) Quantification data of the ratio of preserved motor neurons in each group, data expressed as mean ± SD, ^*^P < 0.001 versus the sham group. ^&^*P* < 0.001 versus the SCI group. *n* = 5 per group.

To investigate the treatment effect of resveratrol on the acute SCI, rats were administered with resveratrol by intraperitoneal injection. BBB rating scale and the inclined plane test were used to assess the motor function of hind limbs at 1, 3, 7, 14, 21 and 28 days after the acute SCI in SD rats. This finding revealed that the BBB scores of the resveratrol group were statistically higher than that those of the SCI group at 7, 14, 21 and 28 days after injury, while no significant difference was found at 1 and 3 days after injury (Figure 1A). Similarly, the inclined plane test scores of the resveratrol group were consistently higher at 7, 14, 21 and 28 days after injury, while no significant difference was found at 1 and 3 days after injury (Figure 1B). These results indicated that resveratrol was capable of improving the motor function of hind limbs after acute SCI in SD rats.

To investigate the effects of resveratrol on motor neurons of the spinal cord after acute SCI, Nissl staining was employed to assess the number of the preserved motor neurons in the spinal cord anterior horn (Figure 1C). As seen from Figure 1D, the number of the preserved motor neurons reduced statistically after SCI, as compared with that in the sham group. However, the number of the preserved motor neurons statistically increased after treatment with resveratrol. These results strongly indicate that resveratrol reduces the loss of motor neurons in the spinal cord anterior horn.

### Resveratrol alleviates the neuronal apoptosis after acute SCI in SD rats

**Figure 2:**
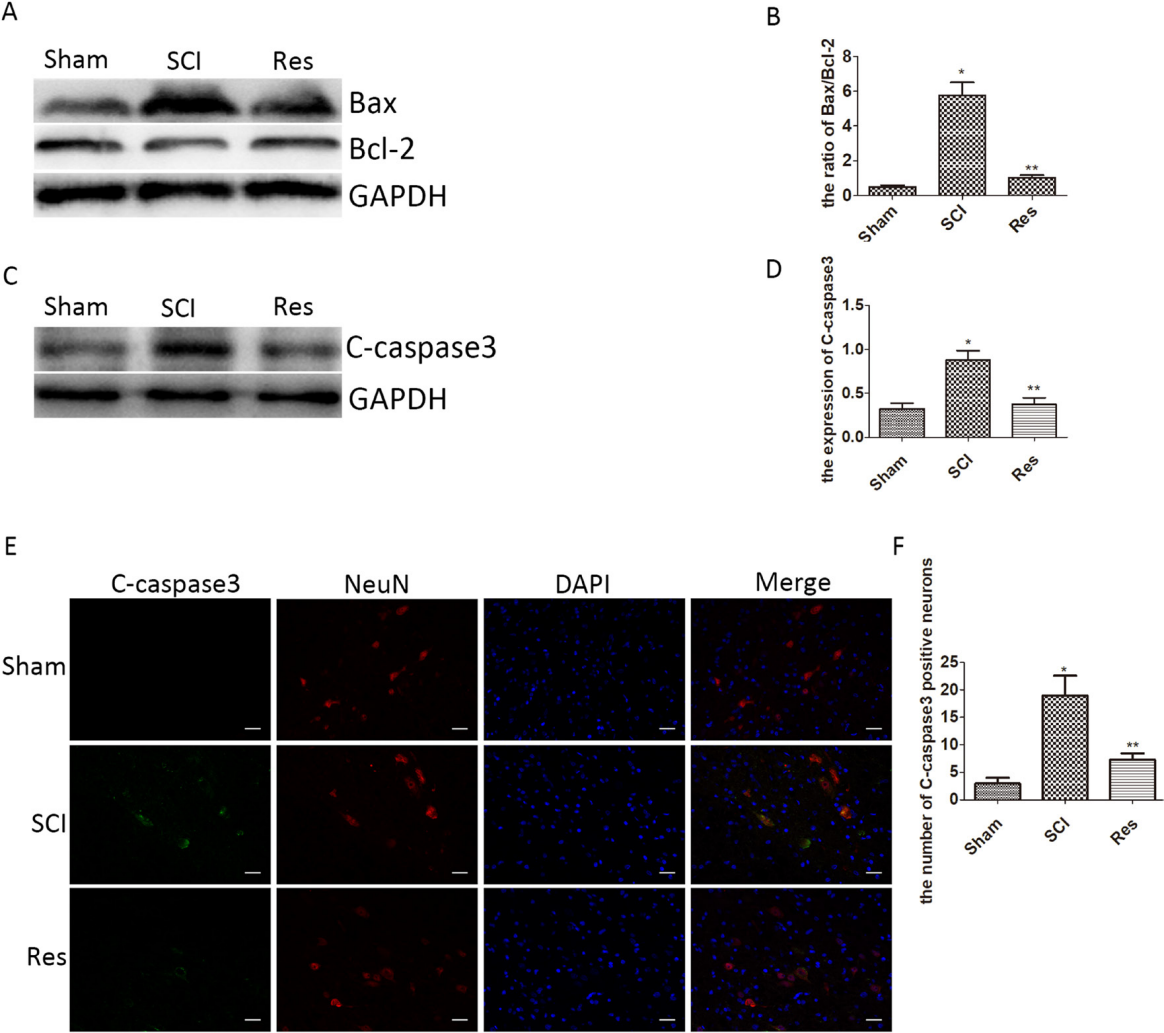
Resveratrol alleviates the neuronal apoptosis after acute SCI in SD rats (**A**) Western blots for the expression of Bax, Bcl-2, and GAPDH in each group. (**B**) Quantification data of the ratio of Bax/Bcl-2 in each group, data expressed as mean ± SD, ^*^P < 0.001 versus the sham group, ^**^P < 0.001 versus the SCI group, *n* = 5 per group. (**C**) Western blots for the expression of C-caspase3 and GAPDH in each group. (**D**) Quantification data of C-caspase3 and GAPDH in each group, data expressed as mean ± SD, ^*^P = 0.01 versus the sham group, ^**^P = 0.006 versus the SCI group, *n* = 5 per group. (**E**) Immunofluorescence of C-caspase3 from the lesion of the spinal cord in the sham, SCI and Res groups in three days after the injury. Scale bars are 20 μm. (**F**) Quantification data of the number of C-caspase3-positive neurons in each group, data expressed as mean ± SD, ^*^P < 0.001 versus the sham group, ^**^P = 0.001 versus the SCI group, *n* = 5 per group.

To confirm the anti-apoptosis effect of resveratrol via our experimental acute SCI model, the apoptosis was assessed in two ways. In the first case, we employed Western blot to demonstrate the level of Cleaved caspase3(C-caspase3), Bcl-2 associated x protein (Bax) and B cell lymphoma/leukemia-2 (Bcl-2). As is shown in Figure 2A, 2B, 2C, 2D, the levels of C-caspase3 and Bax remarkably increased and Bcl-2 remarkably decreased in the SCI group, in contrast to that in the sham group. However, the alteration was reversed in the resveratrol group, in comparison with the SCI group. Moreover, the ratio of Bax/Bcl-2 was significantly increased in the SCI group, as compared with that in the sham group, but the ratio of Bax/Bcl-2 reduced in the resveratrol group as compared with that in the SCI group. In the second case, we employed immunofluorescence staining to evaluate the expression of C-caspase3 in neurons after SCI. As seen from Figure 2E, 2F, the immunofluorescence double staining results also showed that the number of C-caspase3-positive neurons in the SCI group significantly exceeded that in the sham group, while the treatment by resveratrol strongly reduced the number of C-caspase3-positive neurons. This finding proves that resveratrol alleviated the neuronal apoptosis after acute SCI in SD rats.

### Resveratrol ameliorates neuronal autophagic flux after acute SCI in SD rats

**Figure 3:**
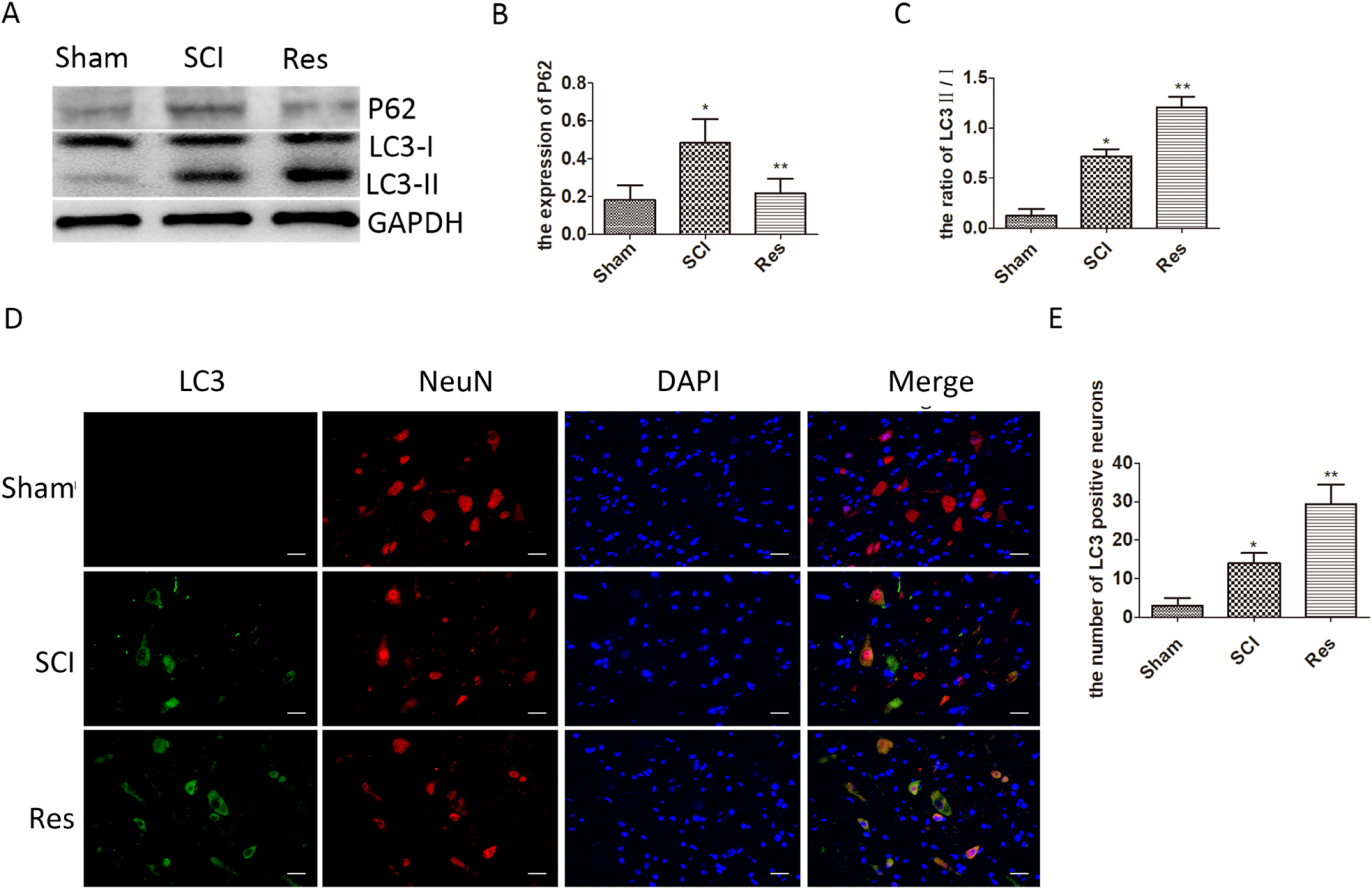
Resveratrol ameliorates neuronal autophagic flux after acute SCI in SD rats (**A**) Western blots for the expression of p62, LC3, and GAPDH in each group in three days after SCI. (**B**) Quantification data of p62 in each group, data expressed as mean ± SD, ^*^P = 0.009 versus the sham group, ^**^P = 0.014 versus the SCI group, *n* = 5 per group. (**C**) Quantification data of LC3-II/I in each group, data expressed as mean ± SD, ^*^P = 0.003 versus the sham group, ^**^P < 0.001 versus the SCI group, *n* = 5 per group(**D**) Immunofluorescence of LC3 from the lesion of spinal cord in the sham, SCI and Res groups in 3 days after SCI. Scale bars are 20 μm. (**E**) Quantification data of the number of LC3-positive neurons in each group, data expressed as mean ± SD, ^*^P = 0.009 versus the sham group, ^**^P = 0.002 versus the SCI group, *n* = 5 per group.

To evaluate the effects of resveratrol on autophagy after acute SCI in rats, the Western blots were used to detect the conversion of microtubule-associated protein 1 light chain 3 (LC3)-I to LC3-II, which was an autophagic marker during autophagosome formation [31]. As illustrated in Figure 3A and 3C, the ratio of LC3-II/I is significantly elevated after SCI, in contrast with the sham group, while the ratio of LC3-II/I is even higher in the resveratrol group. A similar trend was observed by the immunofluorescence of LC3 in neurons (Figure 3D and 3E), indicating that SCI enhanced neuronal autophagy, and resveratrol further promoted neuronal autophagy after acute SCI in SD rats. p62/Sequestosome-1 (p62/SQSTM1, hereinafter referred to as p62) was analyzed by Western blots. As a substrate of the autophagic process, p62 accumulation was related to the obstructed autophagic degradation, whereas p62 was regarded as an autophagic flux marker [32]. The results obtained strongly indicate that the level of p62 was significantly elevated after SCI, in contrast to the sham group, but the level of p62 in the resveratrol group was significantly lower than that in the SCI groups (Figure 3A, 3B). These results confirm that resveratrol ameliorates the neuronal autophagic flux, which is obstructed after SCI in SD rats.

### Resveratrol alleviates the neuronal apoptosis via ameliorating autophagic flux after acute SCI in SD rats

**Figure 4:**
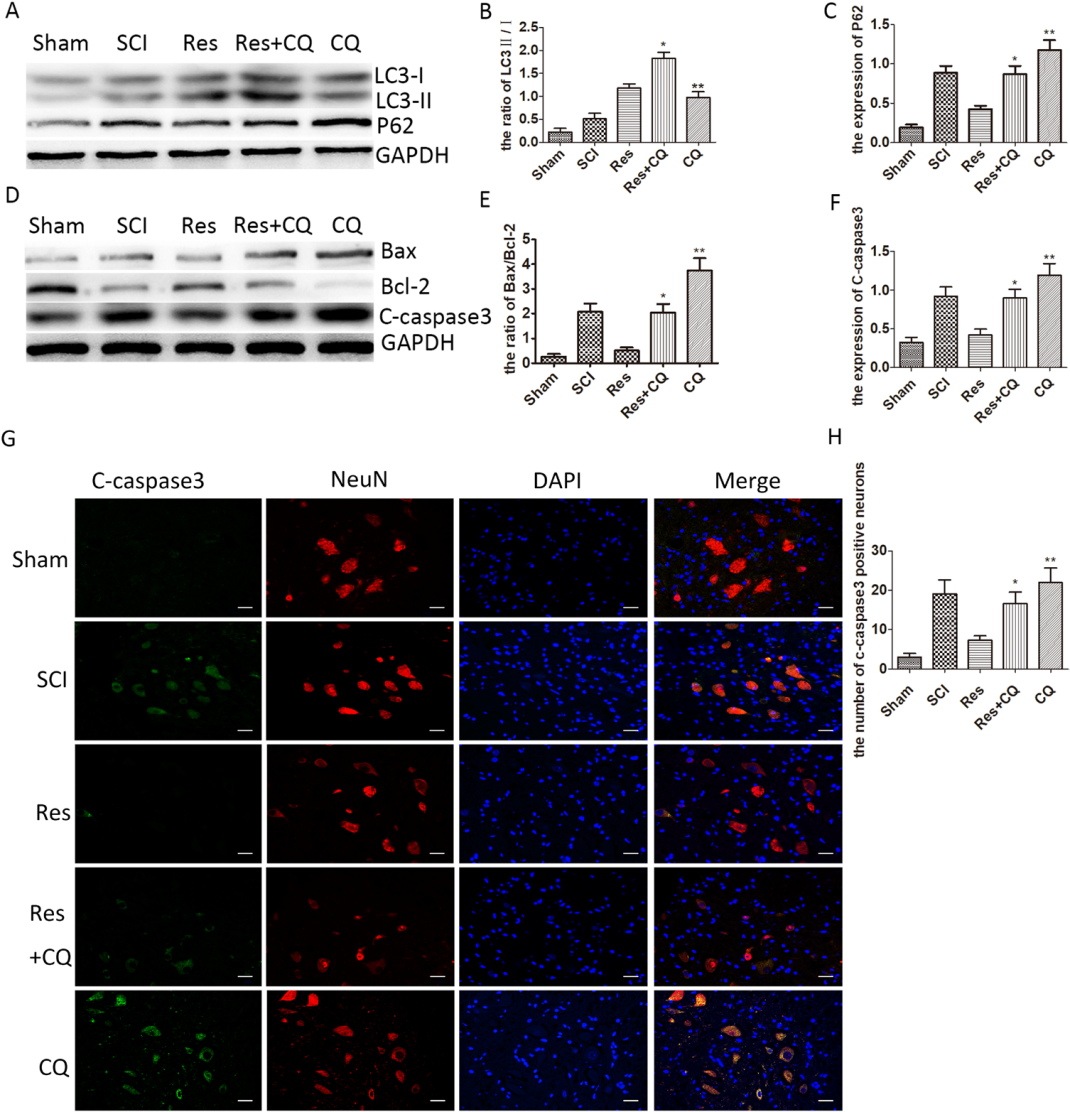
Resveratrol alleviates the neuronal apoptosis by ameliorating autophagic flux after acute SCI in SD rats (**A**) Western blots for the expression of p62, LC3, and GAPDH in each group in three days after SCI. (**B**), (**C**) Quantification data of the ratio of LC3-II/I and p62 in each group, data expressed as mean ± SD, ^*^P < 0.001 versus the Res group, ^**^P < 0.001 versus the Res+CQ group. *n* = 5 per group. (**D**) Western blots for the expression of Bax, Bcl-2, C-caspase3, and GAPDH in each group in three days. (**E**), (**F**) Quantification data of the ratio of Bax/Bcl-2 and C-caspase3 in each group, data expressed as mean ± SD, ^*^P < 0.001 versus the Res group, ^**^P < 0.001 versus the Res+CQ group. *n* = 5 per group. (**G**) Immunofluorescence of C-caspase3 from the lesion of the spinal cord in the sham, SCI, Res, Res+CQ, and CQ groups in three days after SCI. Scale bars are 20 μm. (**H**) Quantification data of the number of C-caspase3-positive neurons in each group, data expressed as mean ± SD, ^*^P =0.002 versus the Res group, ^**^P =0.037 versus the Res+CQ group, *n* = 5 per group.

As observed above, resveratrol significantly influenced the neuronal apoptosis, autophagic flux and the prognosis of acute SCI in SD rats. However, to clarify whether resveratrol mediates the effect of anti-apoptosis through enhancing autophagic flux, chloroquine phosphate (CQ), as an autophagy-lysosomal pathway inhibitor, was administered to inhibit the autophagic flux pathway. As seen from Figure 4A and 4B, the ratio of LC3-II/I was significantly higher in the resveratrol+CQ group than that in the resveratrol group and the CQ group. Meanwhile, the level of p62 was much higher in the resveratrol+CQ group than that in the resveratrol group, while the level of p62 was considerably lower in the resveratrol+CQ group than that in the CQ group. This implies that CQ inhibited the autophagic flux mediated by resveratrol after acute SCI in SD rats. Moreover, the level of C-caspase3 and the ratio of Bax/Bcl-2 increased in the CQ and resveratrol+CQ groups, as compared with that in the resveratrol group (Figure 4D, 4E and 4F). The immunofluorescence double staining results also showed that the number of C-caspase3-positive neurons significantly increased in the CQ and resveratrol+CQ groups, as compared with that in the resveratrol group (Figure 4G and 4H). These results imply that CQ can eliminate the effect of the anti-apoptosis of resveratrol in neurons, and strongly suggest that resveratrol has the capability of alleviating neuronal apoptosis via ameliorating autophagic flux after acute SCI in SD rats.

### Blocking autophagic flux pathway abrogates the effect of preserving neurons and functional recovery mediated by resveratrol treatment after acute SCI in SD rats

**Figure 5:**
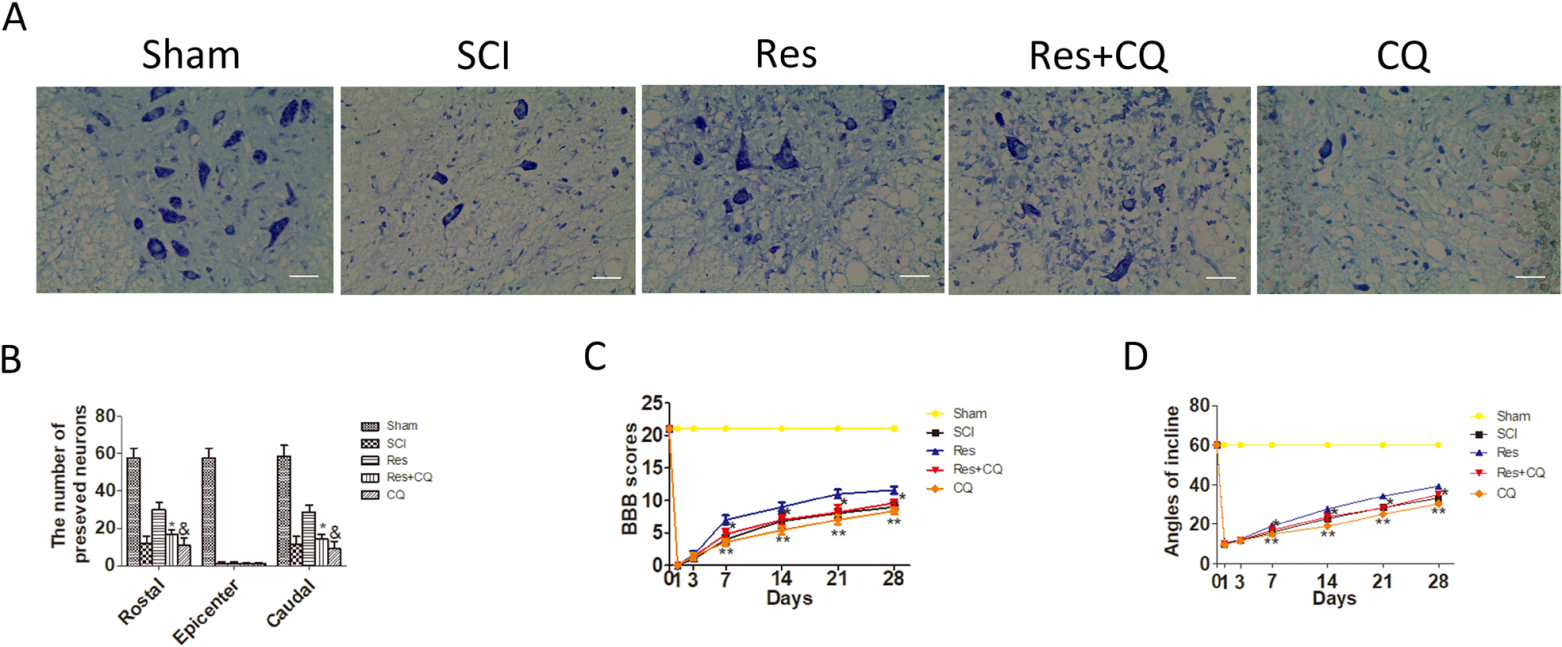
Blocking autophagic flux pathway abrogates the effect of preserving neurons and functional recovery mediated by resveratrol treatment after acute SCI in SD rats (**A**) Nissl staining of sham, SCI, Res, Res+CQ, and CQ groups in 7 days after SCI. Scale bars are 20 μm. (**B**) Quantification data of the number of motor neurons in each group, data expressed as mean ± SD, ^*^P < 0.001 Res+CQ group versus Res group, ^&^ *P* = 0.031 CQ group versus Res+CQ group, *n* = 5 per group. (**C**) The BBB scores of rats in the sham, SCI, Res, Res+CQ, and CQ groups in 1, 3, 7, 14, 21, and 28 days after the injury. ^*^P < 0.001 Res+CQ group versus Res group, ^**^P = 0.007, 0.002, 0.018 and 0.001 CQ group versus Res+CQ group in 7, 14, 21, and 28 days after the injury, respectively. *n* = 5 per group. (**D**) The inclined plane test scores in the sham, SCI, Res, Res+CQ, and CQ groups in 1, 3, 7, 14, 21, and 28 days after the injury. ^*^P < 0.001 Res+CQ group versus Res group, ^**^P < 0.001 CQ group versus Res+CQ group, *n* = 5 per group. Values are expressed as the mean± SD, *n* = 5 per group.

Nissl staining was employed to assess the number of the preserved motor neurons in the spinal cord anterior horn again. As is seen in Figure 5A and 5B, the number of the preserved motor neurons of the resveratrol group was more than that of the CQ combined with or without resveratrol group. These results indicate that CQ partly abrogates the effect of the preserved motor neurons mediated by resveratrol.

To examine whether the enhanced autophagic flux was involved in the treatment effects of resveratrol on improving the motor function of hind limbs, the ethological tests were employed again. As seen in Figure 5C and 5D, both BBB scores and inclination angles of the resveratrol group were statistically higher than those of the CQ and resveratrol+CQ groups. The above data analysis strongly indicates that CQ significantly blocked the effect of improving the motor function of hind limbs mediated by the resveratrol treatment within 7–28 days after acute SCI.

### Resveratrol further activated the LKB1/AMPK/mTOR/p70s6k pathway after acute SCI in SD rats

**Figure 6:**
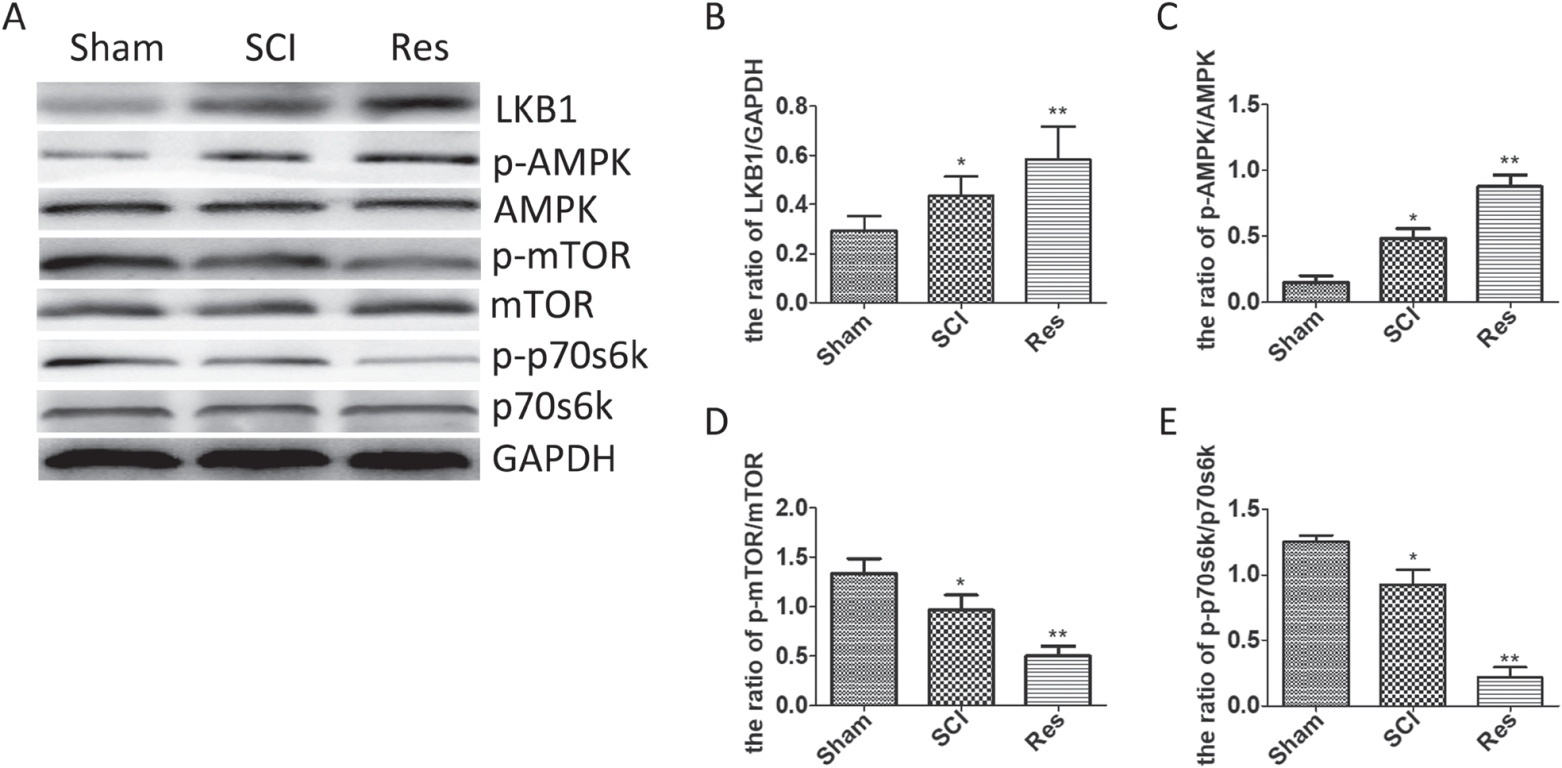
Resveratrol modulated the LKB1/AMPK/mTOR/p70s6k pathway after acute SCI (**A**) Western blots for the expression of LKB1, AMPK, p-AMPK, mTOR, p-mTOR, p70s6k, p-p70s6k and GAPDH in each group in three days after the injury (**B**) Quantification data of the ratio of LKB1/ GAPDH in each group, data expressed as mean ± SD, ^*^P = 0.038 versus the sham group, ^**^P = 0.032 versus the SCI group. (**C**) Quantification data of the ratio of p-AMPK/AMPK in each group, data expressed as mean ± SD, ^*^P < 0.001 versus the sham group,^**^P = 0.001 versus the SCI group. (**D**) Quantification data of the ratio of p-mTOR/mTOR in each group, data expressed as mean ± SD, ^*^P = 0.017 versus the sham group, ^**^P = 0.006 versus the SCI group. (**E**) Quantification data of the ratio of p-p70s6k/ p70s6k in each group, data expressed as mean ± SD, ^*^P = 0.003 versus the sham group, ^**^P < 0.001 versus the SCI group. *n* = 5 per group.

The LKB1/AMPK/mTOR/p70s6k pathway is known to be involved in numerous pathological procesess and to play the main role in the autophagy regulation. In order to investigate whether the LKB1/AMPK/mTOR/p70s6k pathway participated in the autophagy regulation, in case of acute SCI in SD rats, we employed Western blots to examine the level of LKB1, AMPK, p-AMPK, mTOR, p-mTOR, p70s6k, and p-p70s6k. In contrast with the sham group, LKB1/GAPDH and p-AMPK/AMPK was increased statistically in the SCI group and further increased in the resveratrol group (Figure 6A, 6B and 6C). However, the ratios of p-mTOR/ mTOR and p-p70s6k/p70s6k were decreased statistically in the SCI group and further decreased in the resveratrol group (Figure 6A, 6D and 6E). This finding strongly suggests that resveratrol has a pronounced effect on the LKB1/AMPK/mTOR/p70s6k pathway in the acute SCI in SD rats.

### Resveratrol enhances autophagic flux via the LKB1/AMPK/mTOR/p70s6k pathway in PC12 cells

**Figure 7:**
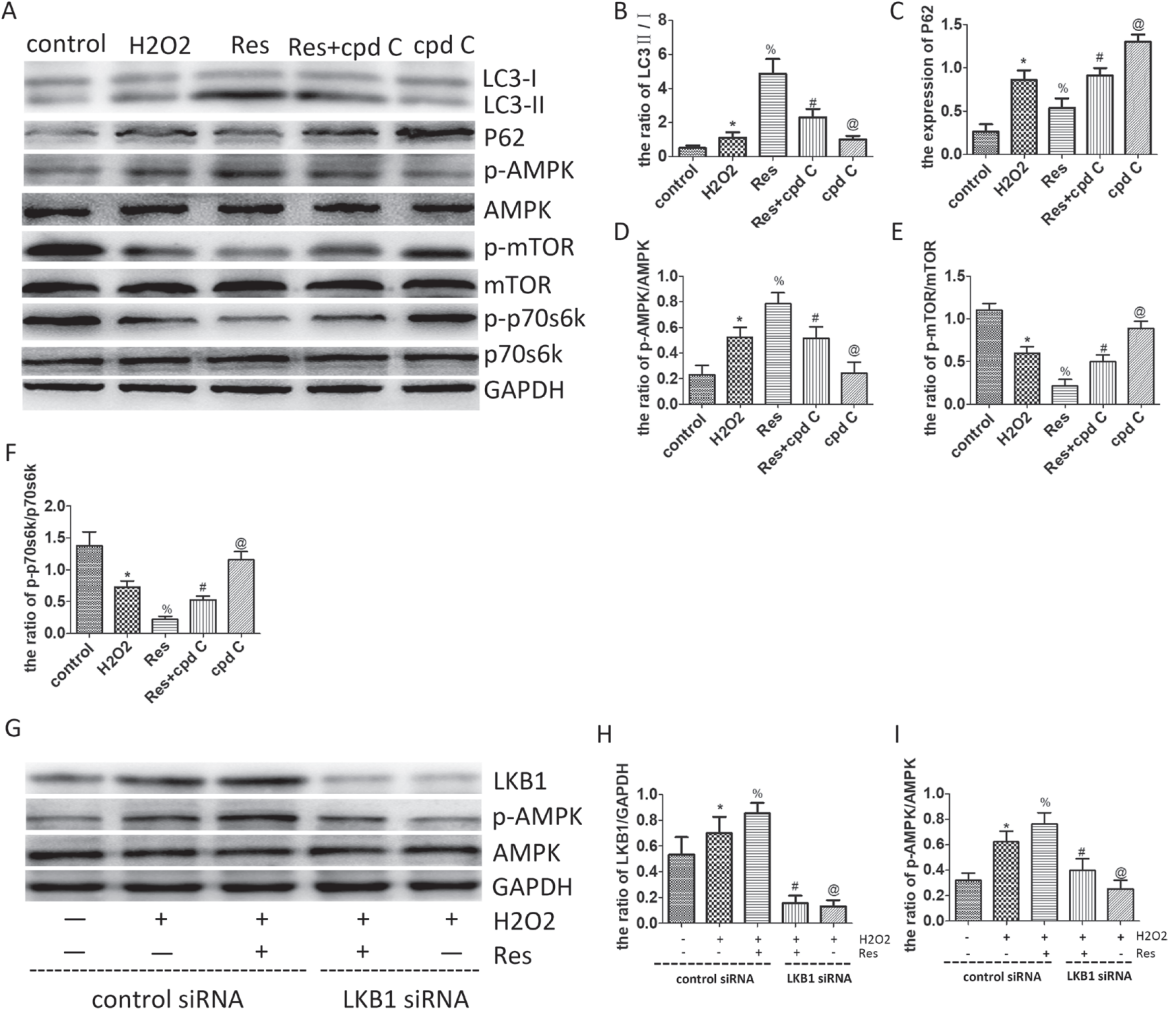
Resveratrol enhances autophagic flux via the LKB1/AMPK/mTOR/p70s6k pathway in PC12 cells (**A**) Western blots for the expression of LC3, p62, AMPK, p-AMPK, mTOR, p-mTOR, p70s6k, p-p70s6k and GAPDH in each group in PC12 cells. (**B**) Quantification data of the ratio of LC3-II/I in each group, data expressed as mean ± SD, ^*^P = 0.049 versus the control group, % *P* < 0.001 versus the H2O2 group, ^#^*P* < 0.001 versus the Res group, ^@^*P* =0.001 versus the Res+cpd C group. (**C**) Quantification data of the ratio of p62/GAPDH in each group, data expressed as mean ± SD, ^*^P < 0.001 versus the control group, % *P* < 0.001 versus the H2O2 group, # *P* < 0.001 versus the Res group, ^@^*P* < 0.001 versus the Res+cpd C group. (**D**) Quantification data of the ratio of p-AMPK/AMPK in each group, data expressed as mean ± SD, ^*^P < 0.001 versus the control group, ^%^*P* < 0.001 versus the H2O2 group, ^#^*P* < 0.001 versus the Res group, ^@^*P* < 0.001 versus the Res+cpd C group. (**E**) Quantification data of the ratio of p-mTOR/mTOR in each group, data expressed as mean ± SD, ^*^P < 0.001 versus the control group, ^%^*P* < 0.001 versus the H2O2 group, # *P* < 0.001 versus the Res group, ^@^*P* < 0.001 versus the Res+cpd C group. (**F**) Quantification data of the ratio of p-p70s6k/ p70s6k in each group, data expressed as mean ± SD, ^*^P < 0.001 versus the control group, ^%^*P* < 0.001 versus the H2O2 group, ^#^*P* = 0.006 versus the Res group, ^@^*P* < 0.001 versus the Res+cpd C group. (**G**) Western blots for the expression of LKB1, AMPK, p-AMPK, and GAPDH in each group in PC12 cells. (**H**) Quantification data of the ratio of LKB1/GAPDH in each group, data expressed as mean ± SD, ^*^P = 0.012 versus the control group, ^%^*P* = 0.021 versus the H2O2 group, ^#^*P* < 0.001 versus the Res+H2O2 group, ^@^*P* = 0.698 versus the Res+LKB1 siRNA+H2O2 group. (**I**) Quantification data of the ratio of p-AMPK/AMPK in each group, data expressed as mean ± SD, ^*^P < 0.001 versus the control group, % P = 0.013 versus the H2O2 group, ^#^*P* < 0.001 versus the Res+H2O2 group, ^@^*P* = 0.01 versus the Res+LKB1 siRNA+H2O2 group. *n* = 5 per group.

To confirm that the AMPK/mTOR/p70s6k pathway was involved in the enhanced autophagic flux mediated by the resveratrol *in vitro*, neuronal PC12 cells were subjected to H2O2-induced oxidative damage, and then resveratrol and compound C (cpd C, the classic AMPK inhibitor) were administered to treat the H2O2-treated-PC12 cells. Western blots were employed to investigate the expression of LC3, p62, AMPK, p-AMPK, mTOR, p-mTOR, p70s6k, and p-p70s6k. As seen in Figure 7A, 7B and 7C, the ratio of LC3-II/I and the level of P62 were upregulated remarkably in the H2O2 group, as compared with that in the control group. This result implies that autophagic flux was obstructed after H2O2 has been administered. Furthermore, the ratio of LC3-II/I was further increased, and the level of P62 was decreased after treatment with resveratrol; therefore, the results showed that the H2O2-induced obstructed autophagy flux was ameliorated by resveratrol treatment. Moreover, the ratio of LC3-II/I was significantly higher, and the level of P62 was much lower in the resveratrol group than those in the resveratrol+cpd C group, where compound C was used in combination with resveratrol or without it (in the cpd C group). This result implies that the resveratrol-induced autophagic flux can be partly eliminated by compound C.

At the same time, the ratio of p-AMPK/AMPK was upregulated remarkably, and the ratios of p-mTOR/mTOR and p-p70s6k/ p70s6k were decreased significantly in the H2O2 group, as compared to the control group. This result implies that the AMPK/mTOR/p70s6k pathway was activated by H2O2-induced oxidative damage. The ratio of p-AMPK/AMPK was further increased remarkably, and the ratio of p-mTOR/mTOR and p-p70s6k/p70s6k were further decreased significantly after treatment with resveratrol. This result indicated that the AMPK/mTOR/p70s6k signaling pathway was further activated by resveratrol after H2O2-induced oxidative damage. However, the ratio of p-AMPK/AMPK was significantly higher, and the ratios of p-mTOR/mTOR and p-p70s6k/p70s6k were considerably lower in the resveratrol group than those in the Res+cpd C and cpd C groups, where compound C was used with or without resveratrol, respectively (Figure 7A, 7D, 7E, 7F). This result implies that the effect of activating AMPK/mTOR/p70s6k pathway mediated by resveratrol was partially or totally suppressed by compound C. These results prove that resveratrol can enhance autophagic flux via the AMPK/mTOR/p70s6k pathway under H2O2-induced oxidative damage in PC12 cells.

To further examine whether activation of AMPK by resveratrol is LKB1-dependent, neuronal PC12 cells were subjected to H2O2-induced oxidative damage, and resveratrol, LKB1 siRNA, and control siRNA were administered to treat the H2O2-treated-PC12 cells. Western blot was employed to investigate the expression of LKB1 and AMPK. As seen from Figure 7G, 7H and 7I, the level of LKB1 and the ratio of p-AMPK/AMPK were increased significantly after treatment with resveratrol. This result indicates that the LKB1/AMPK signaling pathway was activated by resveratrol after H2O2-induced oxidative damage. However, the ratio of p-AMPK/AMPK was significantly higher in the resveratrol group than that in the LKB1 siRNA combined with or without resveratrol group after expression of LKB1 was suppreesed by transfecting PC12 with LKB1 siRNA (Figure 7A, 7D, 7E and 7F). This result implies that the effect of activating AMPK mediated by resveratrol was partially or totally suppressed by LKB1 siRNA. A comprehensive analysis of the above findings corroborated the ability of resveratrol to enhance autophagic flux via the LKB1/AMPK/mTOR/p70s6k pathway under H2O2-induced oxidative damage in PC12 cells.

## DISCUSSION

A spinal cord injury (SCI) is a kind of the central nervous system injury disease with a little reversibility and high disability rate, which brings enormous economic pressure on patients and results in the consumption of large social resources. Treatment of SCI has been a difficult problem in the medical field. Recent studies present that resveratrol plays a neuroprotective role in SCI [23], however, the underlying mechanism is still unclear. Moreover, resveratrol could induce AMPK phosphorylation and inhibit mTOR phosphorylation to protect rat primary cortical neurons from glutamate-induced cell death [21]. Furthermore, resveratrol-induced autophagy can protect one against the neurotoxicity caused by prion protein peptides [33]. In this study, we found that resveratrol enables enhancement of autophagic flux via activating the LKB1/AMPK/mTOR/p70s6k pathway to alleviate neuronal apoptosis, and finally improved the motor function of hind limbs after acute SCI in SD rats.

Apoptosis is one of the crucial biological phenomena. Numerous studies have shown that apoptosis plays a pivotal role in SCI, inhibiting apoptosis can be beneficial to the improvement of the motor function of hind limbs [32, 34, 35]. As an anti-apoptosis protein, Bcl-2 blocked the activation of the upstream caspase protease and inhibited the cell apoptosis by interfering with the release of cytochrome C [36]. As an ion channel component of the mitochondrial membrane, Bax protein enhanced the cytochrome C passage through the mitochondrial membrane, as well as activated caspase-9 and caspase-3, thus leading to apoptosis [37]. Thereby, the decreased Bax/Bcl-2 ratio pertains to inhibited apoptosis [38]. Caspase-3 is also assumed to be a marker of apoptosis and the ultimate performer of apoptosis [39]. It was reported that resveratrol can attenuate the MPP+-induced cell apoptosis in SN4741 cells [40]. Resveratrol also exerts an anti-apoptotic effect, which protects one from neonatal hypoxic-ischemic brain injury [41]. In our study, in contrast to the sham group, the ratio of Bax/Bcl-2 and the level of C-caspase3 remarkably increased after SCI. However, they significantly decreased in the resveratrol group compared with the SCI group. Moreover, resveratrol also significantly reduced the SCI-induced up-regulation of the number of C-caspase3-positive neurons. This evidence revealed that apoptosis was involved in SCI, and resveratrol alleviated the neuronal apoptosis after acute SCI in SD rats. Besides, the number of the motor neurons in the spinal cord anterior horn reduced severely, and the BBB score and the angle of incline decreased after SCI. However, the trend was reversed by treatment with resveratrol. The results indicate that resveratrol was capable of ameliorating the number of the motor neurons and improving the motor function of hind limbs after acute SCI in SD rats.

In recent years, more and more attention has been paid to the function of autophagy in SCI. In fact, the beneficial or detrimental function of autophagy may be dependent on the induction or inhibition of autophagic flux after the central nervous system injury, with the unobstructed autophagy flux often leading to cytoprotection and obstructed autophagy flux contributing to cell death [14]. Recent studies have reported that resveratrol can protect the heart by enhancing autophagic flux to ameliorate myocardial oxidative stress injury [42]. Resveratrol also enhanced the oxidized low-density lipoprotein-induced impaired autophagic flux to mediate protective effect in human umbilical vein endothelial cells [43]. The conversion ratio of LC3-I to LC3-II was an autophagic marker during the autophagosome formation. P62, which is also considered a marker of autophagy flux, is a substrate of the autophagic process. An increase in the level of p62 indicates the autophagy flux reduction and vice versa [14, 44]. In this study, we found that the ratio of LC3-II/I and p62 increased in the SCI group, in contrast to the sham group. This means that the accumulated autophagosome increased, while its degradation slowed down, which implies that autophagic flux was obstructed. However, we found that the ratio of LC3-II/I continued to grow, but the level of p62 dropped down after treatment with resveratrol, in contrast with the SCI group. The results obtained strongly suggest that resveratrol restored the neuronal unobstructed autophagy flux after SCI.

A vast evidence has been accumulated, which proves that the unobstructed autophagy flux can exert a neuroprotection via inhibiting apoptosis in acute SCI [32, 45]. In our study, CQ, which can raise lysosomal pH and disrupt the function of lysosome, leading to the accumulation of LC3-II [46], was administered to block the autophagy flux. Firstly, we found that the level of C-caspase3 and the ratio of Bax/Bcl-2 increased in the CQ combined with or without resveratrol group, as compared with that in the resveratrol group only. Moreover, the number of C-caspase3-positive neurons significantly grew in the CQ combined with or without resveratrol group, as compared with that in the resveratrol group. The result suggested that neuronal apoptosis increased after the resveratrol-induced autophagy flux was obstructed by CQ. In other words, the previous effect of resveratrol on alleviating neuronal apoptosis was eliminated by CQ. Also, the previous effects of resveratrol on preserving motor neurons, and improving the motor function of hind limbs were also removed by CQ. Taken together, these results clarified that resveratrol was capable of restoring the neuronal unobstructed autophagy flux to alleviate neuronal apoptosis and preserving motor neurons, and finally improving the motor function of hind limbs after acute SCI in SD rats.

We further identified the underlying mechanism of the resveratrol enhancing autophagic flux *in vitro*. As a frequently-used neuronal cell line [11, 47], neuronal PC12 cells were subjected to H2O2-induced oxidative damage, then administered with resveratrol, and administered with compound C with or without resveratrol. AMPK-mediated autophagy could exert the neuroprotection effect of ischemic preconditioning in the brain [48]. Inhibition of mTOR could promote the autophagy and alleviate the neuronal death [49]. P70S6K, as the downstream protein of mTOR, reflects the activity of the mTOR signaling pathway [50]. Autophagy can be also activated via AMPK/mTOR/p70s6k pathway [51]. Thus, there may be some intrinsic relationships among autophagic flux, resveratrol and the AMPK/mTOR/p70s6k pathway in SD rats with SCI. In this study, the Western blot results have shown that resveratrol can enhance the autophagic flux, upregulate the level of p-AMPK/AMPK and downregulate the ratio of p-mTOR/mTOR and p-p70s6k/p70s6k *in vivo* and vitro. However, the previous enhancing autophagic flux effect of resveratrol attenuated after the AMPK was inhibited by compound C in H2O2-treated PC12 cells. These results further revealed that resveratrol has the capability of enhancing autophagic flux via activating the AMPK/mTOR/p70s6k pathway. Furthermore, previous study has indicated that LkB1 could suppress apoptosis and activate autophagy, and AMPK could mediate this function of LKB1 in esophageal cancer radiotherapy [52]. Resveratrol cannot elevate p-AMPK expression in the absence of LKB1 in HeLa cells, suggesting that LKB1 alone is sufficient to activate the expression of AMPK [53]. In our study, we found that resveratrol can activate the level of LKB1 and p-AMPK/AMPK *in vivo* and in vitro, and LKB1 siRNA transfection significantly inhibited resveratrol-induced AMPK activation, indicating that resveratrol-induced AMPK activation was mainly proceeded by the activation of LKB1 in PC12 cells. Taken together, these results further revealed that resveratrol has the capability of enhancing autophagic flux via activating the LKB1/AMPK/mTOR/p70s6k pathway.

However, there may be some other potential effects of resveratrol, such as anti-inflammatory, which necessitate our further study. Additionally, the absence of primary neurons *in vitro* is one of the limitations of our study, which may require a further investigation.

In conclusion, our results revealed a new mechanism where resveratrol was capable of restoring the neuronal unobstructed autophagic flux via activating the LKB1/AMPK/mTOR/p70s6k pathway to alleviate apoptosis, and finally ameliorating the motor function of hind limbs after acute SCI in SD rats. Thereby, these results are instrumental for a deeper insight into the underlying neuroprotective effects of resveratrol and polyphenols on the autophagic flux.

## MATERIALS AND METHODS

### Reagents

Resveratrol (Res, Sigma, R5010) dissolved in dimethyl sulfoxide (DMSO; Sigma, D2650) and further dissolved in phosphate-buffered saline (PBS). (Chloroquine Phosphate CQ; Aladdin, C129284) dissolved in PBS. Compound C (cpd C, Aladdin, D139352), Fetal bovine serum (FBS; Gibco, 10099-141), Dulbecco’s modified Eagle’s medium and High Glucose (DMEM/High Glucose, Hyclone, sh30022.01B), 0.25% trypsin solution (Hyclone, sh30042.01), penicillin and streptomycin (Sigma, G4664). Lipofectamine 2000 (Invitrogen, 11668-027), RIPA Lysis Buffer (Beyotime, P0013B), Phenylmethanesulfonyl fluoride (PMSF, Beyotime, ST506), BCA protein enhanced assay kit (Beyotime, P0013B), sodium dodecyl sulfate 53 (SDS) - polyacrylamide gel electrophoresis (PAGE) sample buffer (Beyotime, p0015), polyvinylidene difluoride (PVDF) membrane (Millipore, PVDF, 0.22 or 0.45 mm), Immobilon Western HRP substrate (Millipore, WBKLS0100). Rabbit anti-MAP1LC3B(LC3, Novus, NB100-2220), mouse anti-RBFOX3/NeuN(NeuN, Novus, NBP1-92693), rabbit anti-SQSTM1/p62(P62, Abcam, ab109012), mouse anti-Bcl-2 (Immunoway, YM3041), rabbit anti-Bax (Immunoway,YT0456), rabbit anti-cleaved Caspase-3(Cell Signaling, 9664T), rabbit anti-LKB1(Cell Signaling, 3047S), rabbit anti-AMPK (Cell Signaling, 5831T), rabbit anti-p-AMPK (Thr172) (Cell Signaling, 2535T), rabbit anti-mTOR (Abcam, ab32028), rabbit anti-p-mTOR (Abcam, ab137133), rabbit anti-p70s6k (Cell Signaling, 2708T), rabbit anti-p-p70s6k (Thr389)(Cell Signaling, 9234T), rabbit anti-GAPDH (Earthox, E021060-01). Goat anti-rabbit IgG-HRP (Earthox, E030120-01), goat anti-Mouse IgG-HRP (Earthox, E030110-01), goat anti-rabbit FITCAffiniPure (Earthox, E031220-01), goat anti-mouse Cy3 AffiniPure (Earthox, E031610-01).

### Animals

The healthy adult female Sprague-Dawley rats, weighing 200–250 g, were supplied by the Animal Center of the Chongqing Medical University. All the experimental protocols were ratified by the ethics committee of the Chongqing Medical University, are in full conformity with the national health guidelines for the animal care and usages. The 10% choral hydrate (3 ml/kg) was used for anesthesia by intraperitoneal injection. In this study, we did our best to reduce the number of animals involved in this study and relieve their pain.

### Rat model of SCI and drug treatment

The rat model of SCI was performed based on previous studies [50, 54]. All rats had no food or drink for at least 4 hours before operation. They were anesthetized by intraperitoneal injection of 10% chloral hydrate (3.5 ml/kg, i.p.) and fixed in prostrate position on the operating table. Then the hair around the incision was shaved with an electric razor. After sterilization and sterile sheet application, a post middle approach was made on the back centered on the T10 spine, and then the skin and subcutaneous tissue were cut open. Next, bilateral vertebral muscles were separated bluntly, and the bleeding was stopped by compressing. The T9-T11 spines and lamina were removed carefully. And the dura was exposed and maintained its integrity. Moderate damage was prepared by dropping a 10 g impounder from the height of 25 mm onto the T10 level of the spinal cord without breaking the dura. The striking instrument was withdrawn as soon as possible after the strike, and incision closure then followed. The successful model of SCI implies that the hit spinal cord tissue induces congestion and edema, as well as the spastic swing of the tail and the double lower limbs of the rat, and complete paralysis of the double lower limbs. In the sham group, the spinal cord was only exposed but not damaged. After the operation, all rats were placed in separate cages and allowed to eat and drink. The temperature was kept at 25°C. The bladders of each rat were compressed artificially twice per day to assist the bladder voiding. In this study, no significant side effects from drugs, such as increased mortality or infection rates, were found in the animals under study. Rats were randomly divided into the following five groups: the sham group (*n* = 20), SCI group (*n* = 20), resveratrol (Res) group (*n* = 20), resveratrol combined with CQ (Res+CQ) group (*n* = 20), and CQ group (*n* = 20). After the operation, resveratrol (200 mg/kg/day) was immediately dissolved to be administrated to the rats of the resveratrol group by intraperitoneal injection once a day for three days. At the same time, resveratrol (200 mg/kg/day) and CQ(50 mg/kg/day) were administrated to the rats of the resveratrol combined with CQ group by intraperitoneal injection once a day for three days. CQ(50 mg/kg/day) was administrated to the rats of the CQ group by intraperitoneal injection once a day for three days. The same volume of saline was administrated to the sham group and SCI group by intraperitoneal injection once a day for three days.

### PC12 cells culture and drug administration

The PC12 cells were acquired from the Cell Storage Center of Wuhan University (Wuhan, China). Cells were cultured with Dulbecco’s modified Eagle’s medium and high-glucose (DMEM) supplemented with 10 % fetal bovine serum (FBS), 5% heat-inactivated horse serum, and 1% penicillin and streptomycin at 37°C in a humidified atmosphere containing 5% CO_2_. The PC12 cells were pre-treated with resveratrol (Res, 20 μM) for 2 h, or/and compound C (cpd C, 5 μM) for 2 h after H2O2 (100 μM) treatment for another 2 h. As a control group, the cells were pretreated only with DMSO. Then, cells were divided into five groups: control group, H2O2 group, Res group, Res+compound C group and compound C group.

### RNA interference

PC12 cells were transfected with LKB1 siRNA or control siRNA using Lipofectamine 2000 according to the manufacturer’s protocol. After 24 h transfection, the cells were subsequently incubated with or without H2O2 (100 uM) for 2 h. Then the cells were co-incubated with or without resveratrol (20 μM) for another 2 hours, and they were subsequently collected for Western blot analysis.

### Locomotion recovery assessment.

The Basso, Beattie, and Bresnahan (BBB) scores and the angle of incline test, which were employed to evaluate the motor function of hind limbs after acute SCI in SD rats (*n* = 5 per group) on day 1, 3, 7, 14, 21 and 28 after operation by two independent trained examiners who were blind to the group assignment. The BBB scores, the range of which was from 0 to 21 points, were used to assess the joint movement, gait and coordination of the hind limb and fine motion of toes while moving freely for 5 min in an open field. The higher the scores, the better the motor function of the rats. On the other hand, the higher the angle of the incline, recorded as the maximum angle that a rat can keep its stability for 5 s, the better the motor function of the rats (*n* = 5 rats per group). All experiments were performed in triplicate.

### Nissl staining

At 7 days after SCI, rats (*n* = 5 rats per group) were anesthetized with 10 % chloral hydras (3.5 ml/kg, i.p.) again. And heart perfusion was exerted with 4% Polyformaldehyde. Then the focus of the spinal cord (10 mm) was cut off and fixed in 4% Polyformaldehyde for 24h. Afterwards, these specimens were embedded in paraffin for serial transverse sections (4 μm-thick). Three sections from each rat were selected at 5 mm rostral and caudal to the epicenter for Nissl staining. Next, 1 % cresyl violet acetate was utilized to incubating on the sections for Nissl staining according to the manufacturer’s instructions. The positive Nissl cells were observed and counted under a microscope (Leica, Germany). Five random fields of each section were counted, and then the average preserved motor neurons of each group were calculated.

### Immunofluorescence staining

At 3 days after SCI, spinal cord samples were collected (10 mm, containing the contusion epicenter) (*n* = 5 rats per group), fixed with 4% polyformaldehyde and embedded with paraffin for coronal sections. These sections were dewaxed to water in dimethylbenzene and rewatered in gradient alcohol. Then, the normal goat serum was applied to each section for 30 minutes at 37°C. Afterwards, the goat serum was erased, and the primary antibodies: anti-LC3 (1:200), anti-NeuN(1:400), and anti-C-Caspase3(1:200) were incubated on the sections at 4°C for 12h. Phosphate-buffered saline (PBS) was applied to rinse the sections for 5 min*3 times. As suitable secondary antibodies, goat anti-rabbit FITC AffiniPure and goat anti-mouse Cy3 AffiniPure were incubated on the sections in the dark at 37°C for 1h. After PBS was applied to rinse the sections for 5 min*3 times again, DAPI were incubated on the sections for 5 min at the room temperature subsequently. Finally, the sections were rinsed with PBS for 5min*3 times, covered with coverslips and observed with a microscope (Leica, Germany). Five random fields of each section were counted, and then the average double-labeled positive neurons were calculated.

### Western blot analysis

The protein from spinal cord tissues (10mm, containing the contusion epicenter) (*n* = 5 rats per group) which were cut off at 3 days after SCI and PC12 cells were extracted by means of RIPALysis Buffer containing 1% PMSF. Enhanced BCA Protein Assay Kit was applied to measure the concentration of protein. Then, 53 sodium dodecyl sulfate (SDS)-polyacrylamide gel electrophoresis (PAGE) sample loading buffer was blended with the protein samples for boiling 5 minutes. 40 mg of the protein per group underwent electrophoresis in 8–10% SDS-PAGE gels and were transferred onto polyvinylidenedifluoride membrane. Afterwards, 5% nonfat dry milk was used to block the PVDF membranes for 1h and the membranes were incubated with primary antibodies overnight at 4°C as follows: Bax（1:1000), c-Caspase 3（1:1000), Bcl-2(1:1000), p62 (1:1000), LC3(1:1000), LKB1(1:1000), AMPK(1:1000), p-AMPK(1:1000), mTOR (1:1000), p-mTOR (1:1000), p70s6k(1:1000), p-P70s6k(1:1000) and GAPDH (1:1000). Washed with TBST 10minutes*3 times later, the membranes were incubated with second antibodies: goat anti-rabbit IgGHRP (1:10000), goat anti-mouse IgGHRP (1:10000) 1h at 37°C. Washed with TBST 10minutes*3 times later, the membranes were immersed in Immobilon Western HRP substrate and further developed. The bands were quantified and analyzed by the fusion software.

### Statistical analysis

SPSS 17.0 statistical software was used to perform the statistical analysis. Each quantitative analysis was repeated 3 times by use of the same tissues/cells. Results were expressed in terms of means ± standard deviation (SD) for at least three independent experiments. Any statistical significance was calculated employing the unpaired Student’s *t*-test or one-way variance analysis, and *P* value of < 0.05 was considered statistically significant.
